# The Management of Complex Knee Deformity Using Ortho-SUV: A Case Study

**DOI:** 10.7759/cureus.68065

**Published:** 2024-08-28

**Authors:** Kaustubh Zodey, Sushil Mankar, Pallav P Agrawal

**Affiliations:** 1 Orthopedics, N.K.P. Salve Institute of Medical Sciences (NKPSIMS) and Lata Mangeshkar Hospital, Nagpur, IND; 2 Orthopedics and Traumatology, N.K.P. Salve Institute of Medical Sciences and Research Centre (NKPSIMS and RC), Nagpur, IND

**Keywords:** ortho-suv, multiplanar deformity, complex knee deformity, femoral osteotomy, ilizarov fixator

## Abstract

Lower limb deformities are debilitating and affect the function of the lower limb and the social life of the patient. The deformities around the knee joint might be a combination of multiple deformities in various planes including coronal, sagittal, or rotational and may be present in both the femur and the tibia. We present a case of a 19-year-old female with complex deformity around the left knee joint involving both the femur and the tibia with shortening. The deformity was thoroughly planned and managed with both the Ortho-SUV and the Ilizarov technique. Complications such as neurological injury and nonunion were faced, but the neurology was improved spontaneously, and the autologous bone graft was done. The end result was excellent with no residual neurodeficit and no limb length discrepancy. Deformities such as these have to be addressed with meticulous planning and strategic surgical management. Complications such as nonunion, infection, neurological injury, and noncompliance might be faced in the course of the treatment. Though the Ilizarov apparatus is the most commonly used to address deformities and limb lengthening, the newer six-axis devices such as the Ortho-SUV can greatly aid in deformity correction.

## Introduction

Deformities around the knee joint present significant challenges for orthopedic surgeons. These deformities are often diagnosed at advanced stages, where treatment becomes complex, necessitating multiple surgical interventions and potentially leading to complications [1,2]. These deformities typically manifest as combinations of abnormalities in various planes, such as coronal (varus/valgus), sagittal (procurvatum/recurvatum), and rotational (internal/external). When located around the knee joint, deformities may affect the distal femur, proximal tibia, or both [3]. Such deformities disrupt the proper distribution of forces across weight-bearing joints, resulting in functional disturbances and alterations in gait, potentially leading to early degenerative arthritis over time [4]. Deformities in the sagittal and coronal planes can be assessed using full-length X-rays [5]; however, planning for rotational plane deformities poses clinical and radiologic challenges. With thorough clinical and radiologic assessment and precise preoperative planning, accurate correction with favorable functional outcomes can be achieved. Multiple surgical options and techniques are available to address these complex, multiplanar, lower limb deformities using both internal and external fixators [6]. The Ilizarov ring is the most commonly employed apparatus for deformity correction, yielding satisfactory results [7]. However, it sometimes necessitates correction across multiple planes. The Ortho-SUV is a six-axis external fixator [8] that utilizes actual X-ray images to calculate the deformity and provide an adjustment schedule [9]. Herein, we present a unique case of a 19-year-old female with a complex multiplanar deformity of the distal femur and proximal tibia treated with a staged treatment approach, involving plating for the distal femur and Ortho-SUV frame for the proximal tibia.

## Case presentation

**Figure 1:**
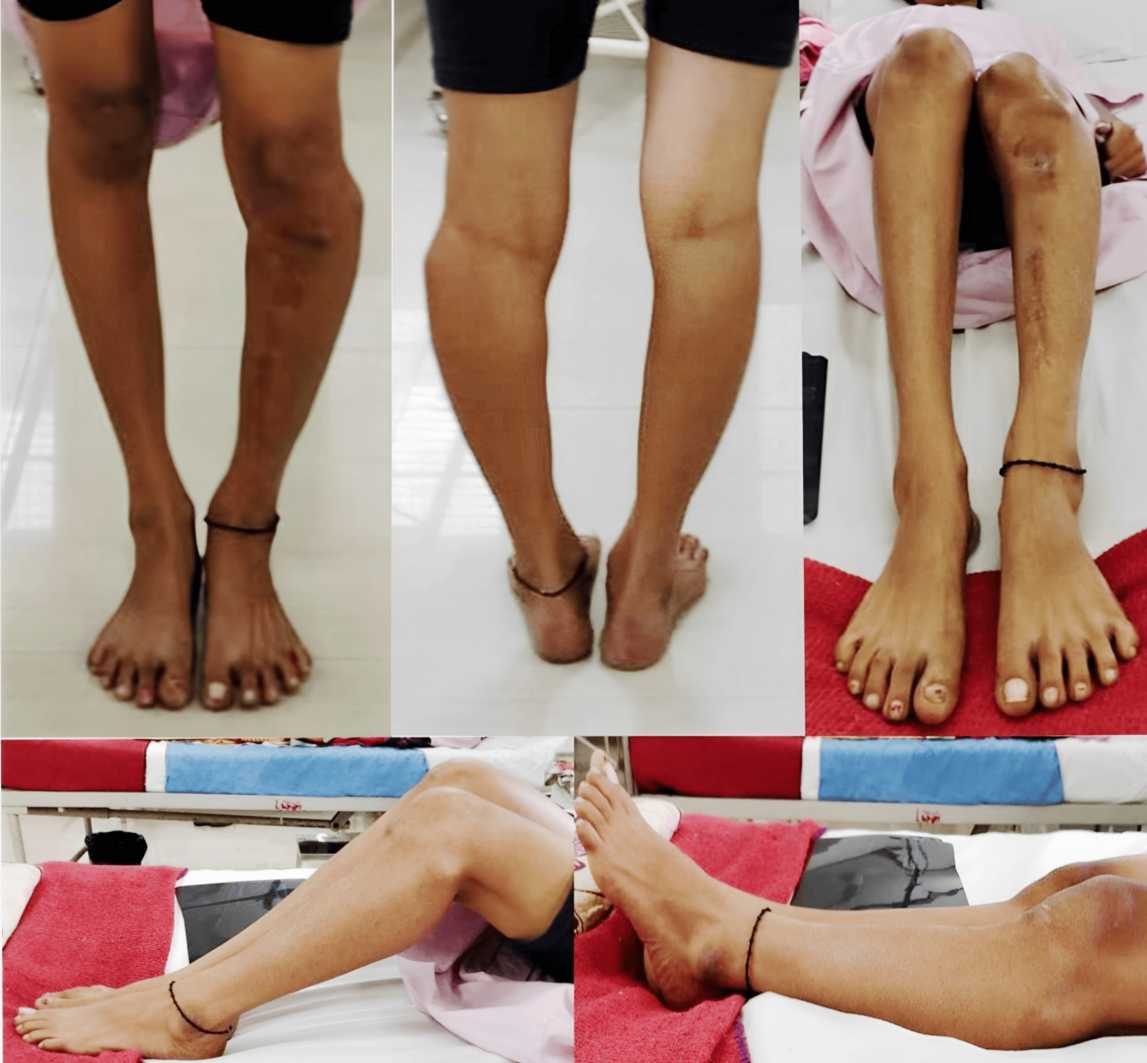
The clinical pictures of the deformity in a standing and supine position, captured from all sides.

A 19-year-old female nursing student presented to our outpatient department with a complex deformity around the left knee joint. The patient exhibited a knee varus deformity accompanied by a shortening of the lower limb. Notably, a 9 cm scar was observed on the anteromedial aspect of the left leg (Figure 1). The patient reported undergoing a surgical procedure 10 years prior due to an infection; however, no documentation of the previous procedure was available.

**Figure 2:**
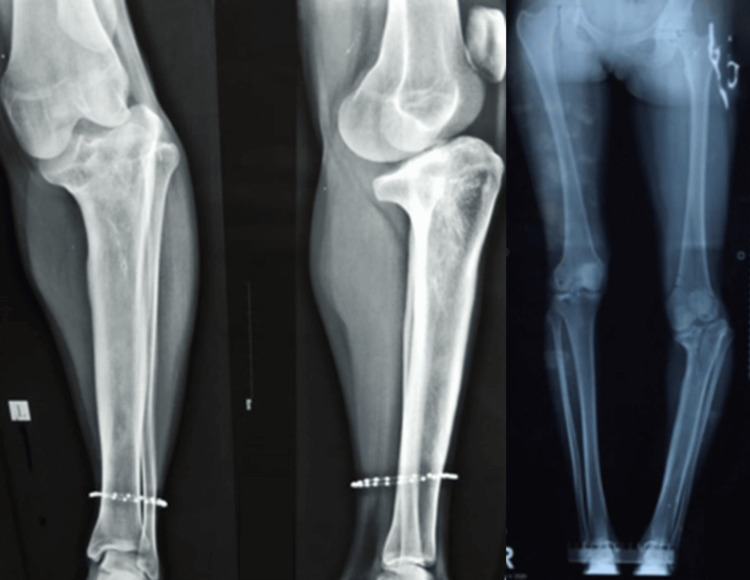
Preoperative X-rays of the aforementioned deformity.

After a thorough clinical examination and measurement, a shortening of 8 cm was noted. No rotational deformity was noted at the hip joint; on examination, the ankle and hip joints were normal and had a full range of motion. Necessary hematologic and radiologic examinations were performed (Figure 2), and the following radiologic parameters were calculated: (1) mechanical axis deviation, 30%; (2) medial proximal tibial angle, 60°; (3) lateral distal femoral angle, 78°; (4) posterior tibial slope (sagittal), 30°; and (5) limb length discrepancy, 8 cm shortening on the left side.

The radiologic examination indicated that the deformity resulted from a varus deformity of the proximal tibia (medial proximal tibial angle of 60°) with a compensatory valgus of the distal femur (lateral distal femoral angle of 78°). Additionally, a tibial slope of 30° was observed in the sagittal plane. The combination of these multiplanar deformities was associated with a shortening of 8 cm.

**Figure 3:**
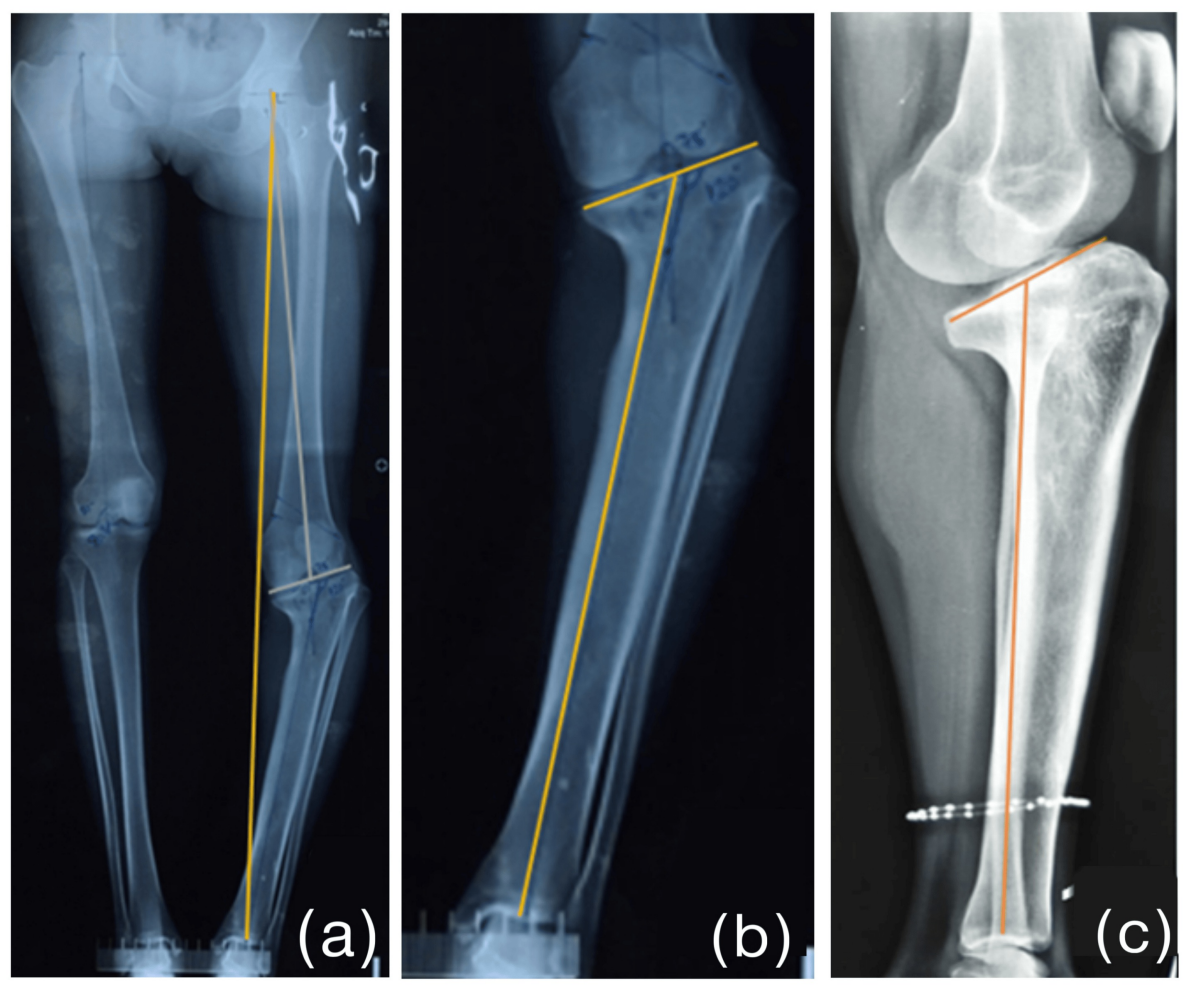
Preoperative images showing mechanical axis deviation. (a) Preoperative planning with mechanical axis deviation in a full-length standing scanogram and the lateral distal femoral angle. (b) The medial proximal tibial angle. (c) The posterior slope.

Following comprehensive preoperative planning (Figure 3) and discussion, a staged procedure with gradual correction was devised. Necessary hematologic investigations were conducted to ensure the patient’s fitness for surgery.

Surgical procedure

**Figure 4:**
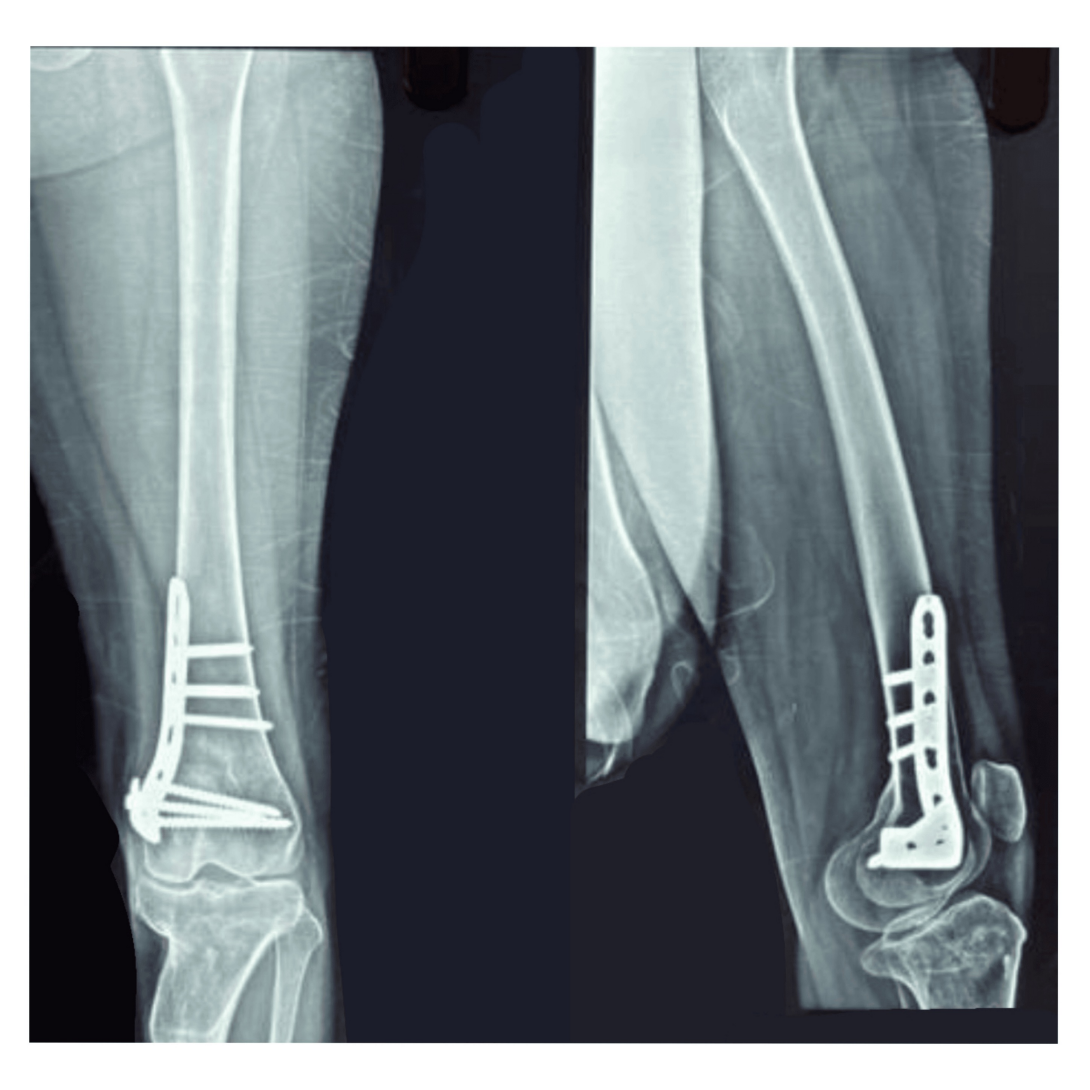
The correction of the distal femur deformity using a closed wedge osteotomy with a plate.

**Figure 5:**
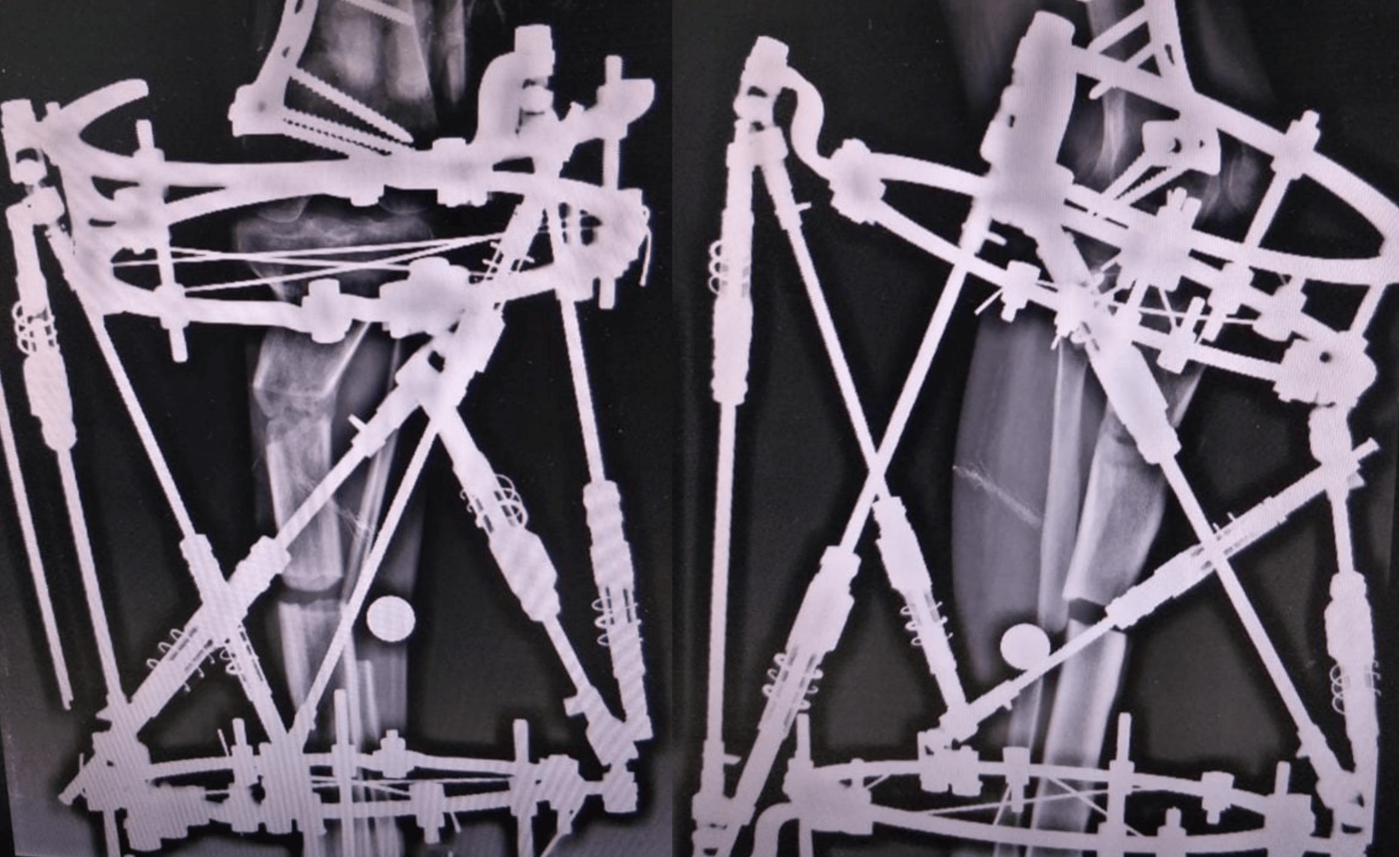
The anteroposterior and lateral views of the deformity correction using the Ortho-SUV fixator.

In the first stage of treatment, the correction of the distal femur deformity was undertaken concurrently with Ortho-SUV for the proximal tibia. A medial closed wedge osteotomy with a medial distal femur locking plate was performed under spinal anesthesia. A 20° wedge was excised, achieving the acute correction of the distal femur deformity (Figure 4). Simultaneously, the deformity of the proximal tibia was addressed. The correction of all the components of the proximal tibia-tibial varus, slope, and shortening was planned using the Ortho-SUV software. After obtaining appropriate X-rays for the Ortho-SUV software, the center of rotation of angulation (CORA) was identified. However, the CORA was found to be very proximal, leaving no space for the most proximal tibial ring. Hence, a corticotomy for correction was performed just distal to the CORA, and correction proceeded according to the software protocol (Figure 5). Following the acute correction of the distal femur deformity, the patient developed a foot drop. Electromyography and nerve conduction studies confirmed neuropraxia, likely due to nerve stretching during acute correction. The patient was strictly non-weight-bearing for 12 weeks, and correction using Ortho-SUV was initiated as per the established protocol.

**Figure 6:**
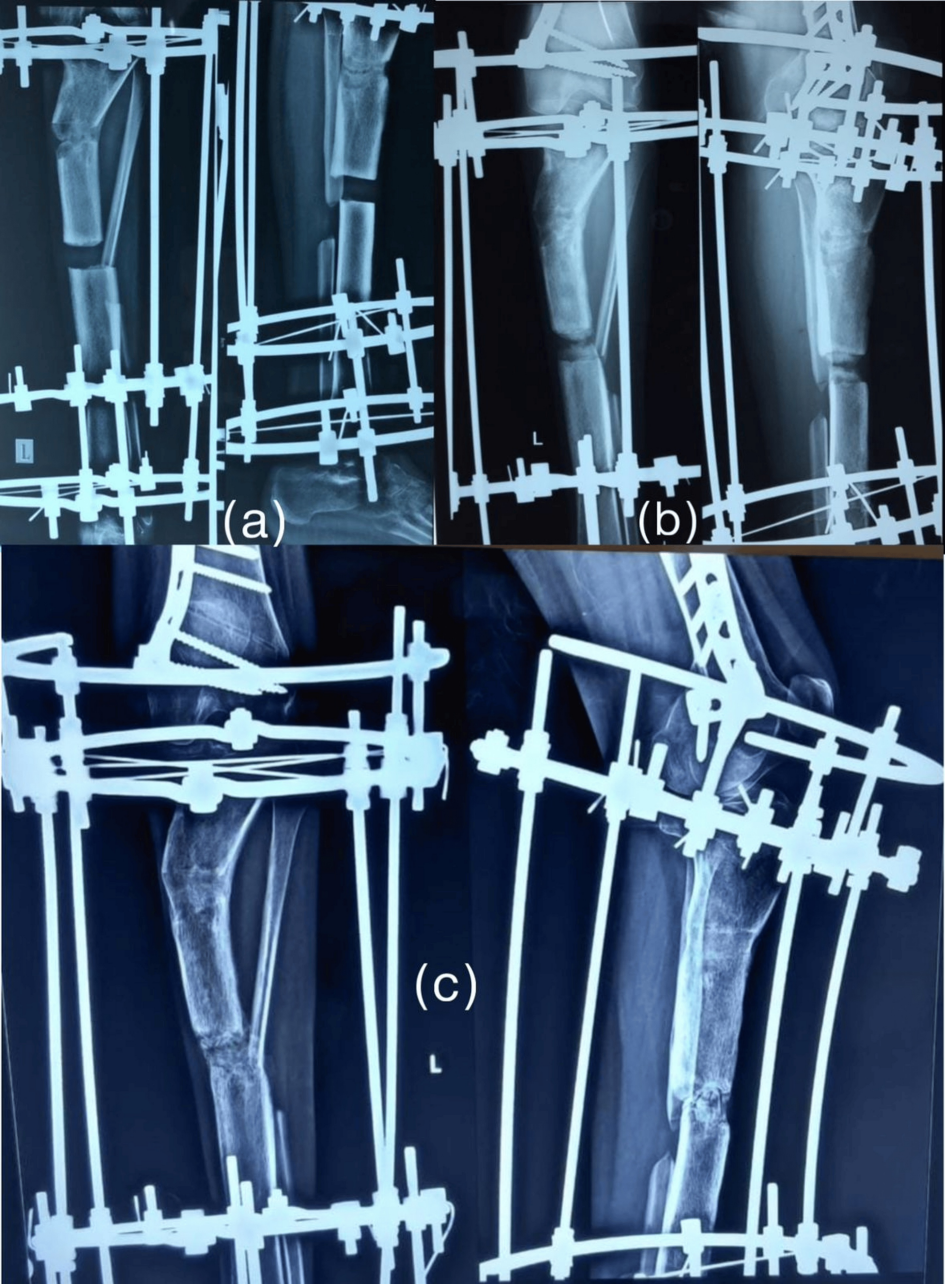
Sequential images of the Ilizarov frame with corticotomy at the tibia shaft, distraction phase, and consolidation phase aided by bone grafting at the distraction site.

The deformity correction was successfully completed using the Ortho-SUV frame. Following the procedure, the neurological deficit spontaneously improved, and complete recovery was observed six weeks after the tibial correction was finalized. However, a residual shortening of 6 cm persisted. To address this, the Ortho-SUV frame was removed, and a revision Ilizarov technique was employed. Corticotomy was performed at a more distal site in the tibial shaft, followed by gradual lengthening through distraction. The calculated distraction continued for three months at a rate of 1 mm per day. Subsequent radiographs revealed inadequate regenerate formation at the distraction site, resulting in nonunion. To promote union, autologous bone grafting was performed at the distraction site. The Ilizarov frame remained in place for 18 months following the final procedure to support stabilization and healing (Figure 6).

**Figure 7:**
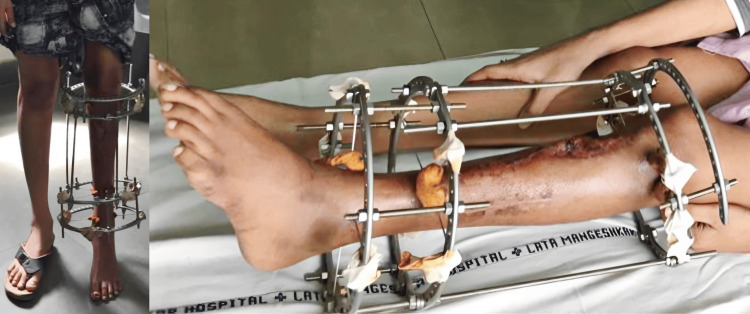
Clinical images illustrating the Ilizarov frame with the completed correction of the deformity without any limb length discrepancy.

After 18 months, the Ilizarov frame was removed following the clinical and radiologic confirmation of union at both the deformity correction site and distraction site (Figure 7).

**Figure 8:**
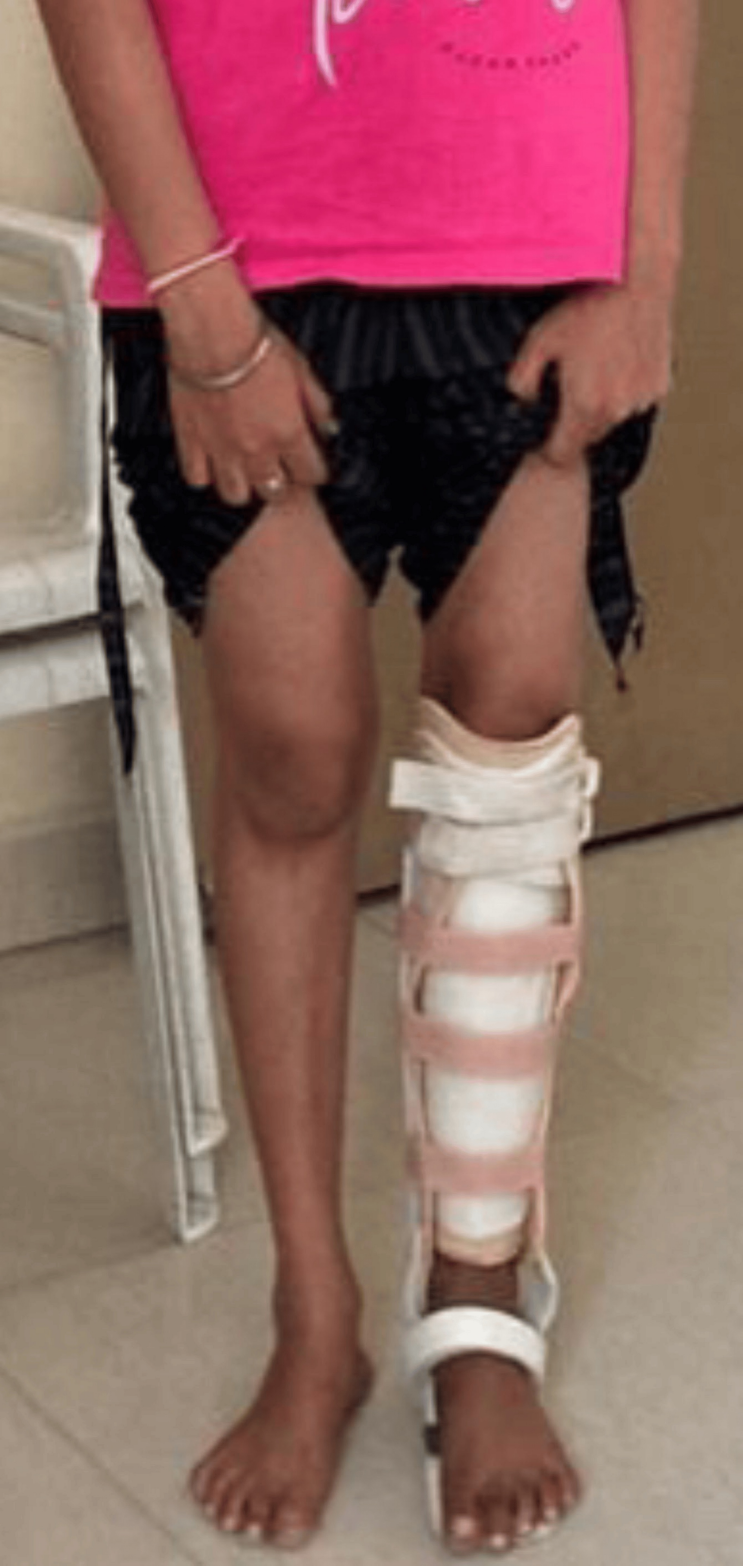
A removable thermoplastic plastic brace following the removal of the Ilizarov frame.

**Figure 9:**
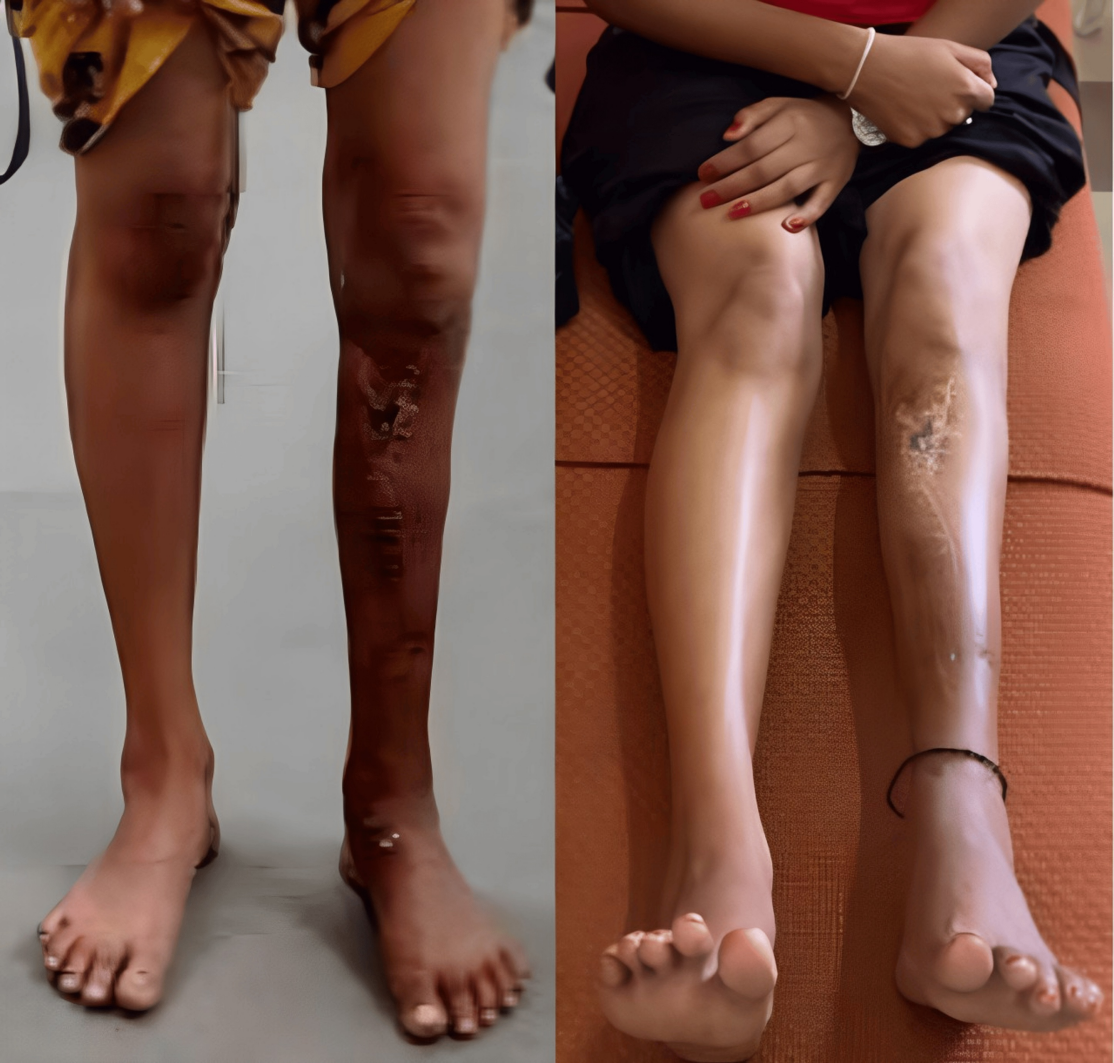
The corrected deformity in all planes with bilateral equal limb lengths.

**Figure 10:**
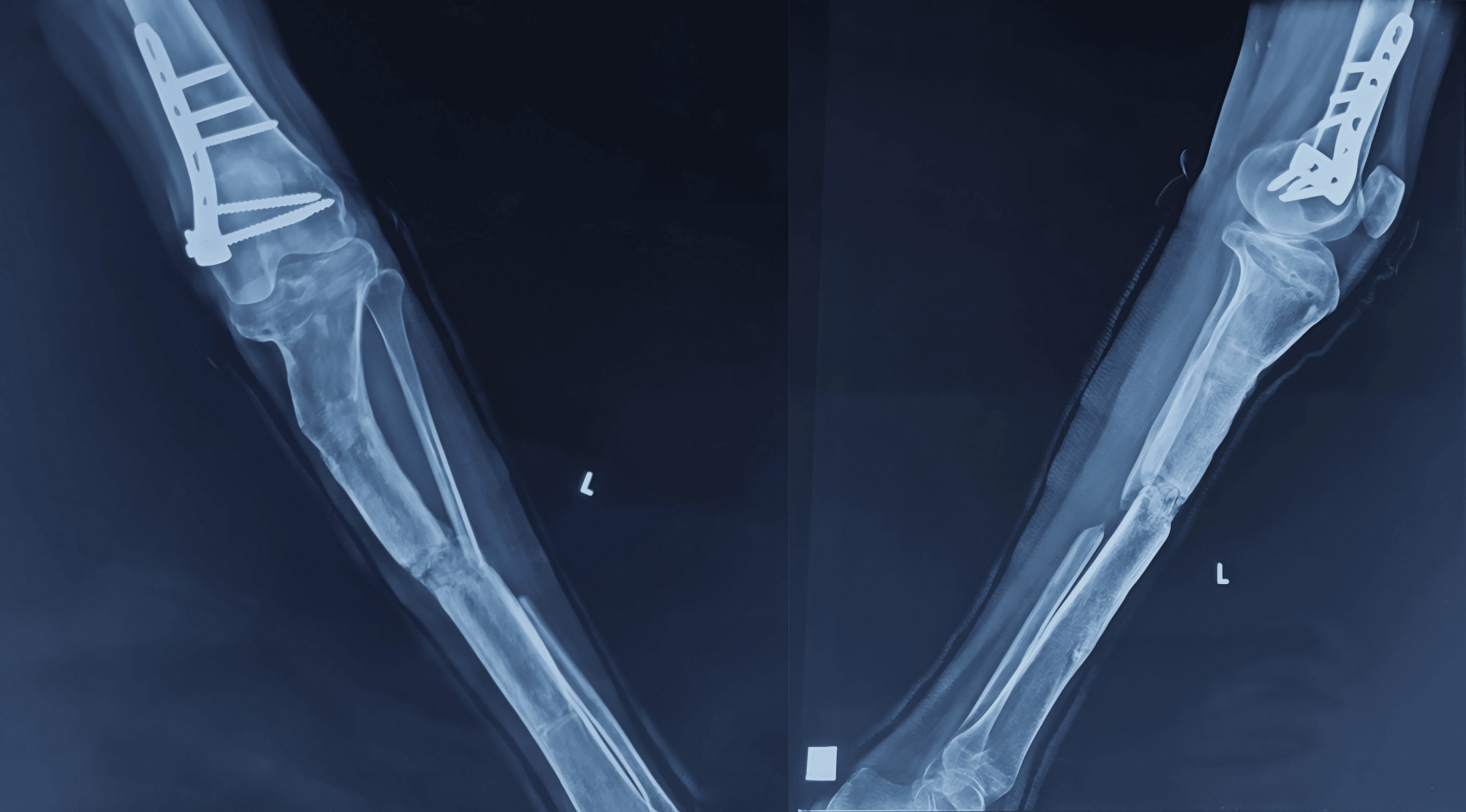
Two-year follow-up postoperative X-ray anteroposterior and lateral views showing good union in the tibia with a significant correction of the deformity.

Subsequently, a customized thermoplastic removable brace was provided, as shown in Figure 8, and worn for 12 months thereafter. Upon the removal of the frame and the use of the brace, the patient was permitted full weight-bearing walking and encouraged to achieve full knee range of motion. Notably, there were no postoperative neurological deficits, and no limb length discrepancies were noted, as depicted in Figure 9. Postoperative X-ray showed good bone union in the tibia with significant deformity correction done at two-year follow-up (Figure 10).

## Discussion

Complex knee deformity represents a disabling condition for patients, particularly among young and adolescent individuals, necessitating corrective measures to improve function and social esteem [10]. While simple deformities around the knee in single planes are manageable, severe and complex deformities associated with limb shortening require meticulous attention from the treating surgeon. Comprehensive preoperative planning is essential for the effective management of these cases and may require multiple surgeries to fully correct the deformity [11]. Correcting deformities across multiple planes can pose risks such as neurological injury and malalignment. Traditionally, the Ilizarov apparatus has been extensively used to address such cases. However, the newly developed external fixator, employing a six-axis correction method such as the Ortho-SUV, now offers a comprehensive correction of all the deformities in both sagittal and coronal planes in a single surgical procedure [8]. This system also allows for the correction of limb shortening. In our case, a young adolescent female presented with complex deformities in both the distal femur and proximal tibia, which were successfully corrected using a plate for the distal femur, the Ortho-SUV for the proximal tibia, and the Ilizarov method to correct limb length discrepancy. During the treatment, the patient experienced a temporary foot drop, which improved spontaneously without any intervention. Postoperatively, the patient demonstrated improved knee function and achieved excellent outcomes, successfully resuming all activities of daily living.

## Conclusions

In conclusion, we believe that the precise correction facilitated by modern techniques such as the Ortho-SUV and gradual correction by the Ilizarov method represent powerful tools for orthopedic surgeons. These approaches consistently yield excellent results without compromising limb length. Nevertheless, long-term follow-up is crucial to monitor bracing and function and to mitigate potential complications for optimal results.
